# The Role of Macrophage Migration Inhibitory Factor in Mast Cell-Stimulated Fibroblast Proliferation and Collagen Production

**DOI:** 10.1371/journal.pone.0122482

**Published:** 2015-03-31

**Authors:** Gu Ningyan, Yao Xu, Shi Hongfei, Chen Jingjing, Chen Min

**Affiliations:** 1 Institute of Dermatology, Chinese Academy of Medical Sciences and Peking Union Medical College, Nanjing, China; 2 Department of Orthopedics, The Affiliated Drum Tower Hospital of Nanjing University Medical School, Nanjing, Jiangsu, China; The Ohio State University, UNITED STATES

## Abstract

Current clinical and translational studies have shown that mast cell plays a pivotal role in multiple fibrotic diseases including scleroderma. However, the lack of mature human mast cell culture model exhibits a major obstacle for further dissection of cytokines and signaling molecules required for mast cell mediated fibrosis in various diseases. Macrophage Migration Inhibitory Factor is a mast cell released pro-inflammatory cytokine which is deregulated in scleroderma patients and is also involved in non-scleroderma related fibrosis. In the current study, we successfully generated a practical and reliable human mast cell culture system with bone marrow CD34+ hematopietic precursors. The derivative mast cell is normal in terms of both morphology and function as manifested by normal degranulation. More importantly, we were able to show mast cell conditioned medium as well as MIF supplementation augments fibroblast proliferation and collagen synthesis. This positive regulatory effect of mast cell conditioned medium can be dampened by MIF antibody. In addition, MIF-knockdown significantly inhibits pro-fibrotic activities of CD34+ hematopietic precursor derived mast cells. These data strongly suggest that mast cell released MIF is required for mast cell mediated fibrogenic activities. The current manuscript seems to be the first mechanistic report showing the significance of MIF in mast cell mediated fibrosis, which may pave the way for the development of potential MIF-targeted therapy for fibrotic diseases to a further extent. Moreover, we strongly believe mast cell culture and differentiation model as well as corresponding genetic manipulation methodology will be helpful in characterizing novel mast cell based therapeutic targets.

## Introduction

Mast cells (MCs) were first described by von Recklinghausen in 1863 [1]. Derived from bone marrow progenitors, MCs can be found at locations in proximity to environment-host interface for their participation in innate immunity. Traditionally, MCs are most well known for their role in IgE-mediated immune responses. Another important feature of MCs is their capability of secreting various mediators, such as histamine, chymase and TGF-β [2]. Although these fascinating cells have attracted remarkable research interest, many aspects of mast cell biology including their origin, development and functions still need further elucidation [3].

Numerous lines of evidence have demonstrated the involvement of mast cells in fibrogenic conditions such as pulmonary fibrosis, liver cirrhosis and renal interstitial fibrosis [4–6]. More importantly, recent studies have revealed that mast cells have multiple functions in pathogenesis and development of scleroderma (systemic sclerosis). As a chronic systematic and heterogeneous autoimmune disease, scleroderma is featured by vascular alterations, autoimmunity and fibrosis. Especially, a distinguishing hallmark of scleroderma is progressive fibrotic replacement in multiple organs with unknown etiology. Alterations of mast cells, including changes in their numbers and functions, have been observed at sites of fibrosis in scleroderma [7–11]. In the study using the tight-skin mouse model of scleroderma, a remarkable increase of mast cell number during fibrosis in the skin lesions was observed [12]. It has been shown that the mast cell-released cytokines contribute to various fibrogenic effects [13, 14]. Using human mast cell line HMC-1, Garbuzenko et al showed that human mast cells stimulate fibroblast proliferation, collagen synthesis and lattice contraction [15]. More specifically, several studies have showed that mast cell-derived cytokines, including chymase and TGF-β which have pro-fibrotic activities [16, 17], are up-regulated in the affected skin of scleroderma [12, 18, 19]. Along this line, inhibition of mast cell-derived cytokines has showed therapeutic benefits to scleroderma in mouse models [11, 20].

Among cytokines secreted by mast cells, we are particularly interested in macrophage migration inhibitory factor (MIF). Huaxian *et al*. first discovered that MIF was constitutively expressed at high levels in human mast cells [21]. In 2003, Selvi *et al*. reported that the serum concentrations of MIF in patients with diffuse form of systemic sclerosis were significantly higher than those in controls and MIF expression was detected in skin biopsies of scleroderma patients by immunohistochemical staining [22]. Several independent studies have reported that a functional MIF promoter polymorphism (-173G/C) which often results in high production of MIF was strongly associated with diffuse cutaneous systemic sclerosis [23, 24]. In addition, Becker *et al*. revealed that MIF may contribute to vasculopathy in systemic sclerosis [25]. On the other hand, MIF was reported to facilitate fibrosis process which represents the substantial pathological changes in scleroderma [26–29]. Based on these existing data, we hypothesized that MIF produced by mast cells may promote fibrosis process in pathogenesis of scleroderma.

To further understand the mechanism of scleroderma, it’s important to investigate the functions of MCs in fibrosis. Although significant progresses have been made during the past several decades, major limitations exist in the studies of MCs: first of all, there are significant differences between human and mouse mast cells; secondly, using HMC-1 cells (a cancer cell line) to represent human mast cells may not be always appropriate; finally, although normal human mast cells can be isolated from several sources, it is difficult to perform genetic manipulation on these cells.

Inspired by the innovative studies done by Kovarovam M. et al. [30, 31], we sought to derive MCs from human CD34+ hematopoietic precursors. Our purpose is to provide a large number of normal mast cells for further study in human context and to establish an appropriate genetic manipulation methodology for MCs. More importantly, we examined the effects of MIF, an important cytokine produced and released by MCs, on fibroblast proliferation and collagen production.

## Materials and Methods

### Materials

Dulbecco's Modified Eagle's Medium (DMEM), Iscove’s modified Dulbecco’s medium (IMDM), fetal bovine serum (FBS) and penicillin-streptomycin solution were purchased from Life Technologies (Gibco). L-glutamine, ascorbic acid, 1-thioglycerol, HEPES buffer, nonessential amino acids and sodium pyruvate were purchased from Sigma. Human stem cell factor (SCF), interleukin 6 (IL-6) and interleukin 3 (IL-3) were obtained from Peprotech. Recombinant Human MIF (Z03159-50) was obtained from Genscript. 6-well plates and 10-cm ultra-low-attachment dishes were purchased from Corning. Anti-chymase (MA5-11717) and anti-tryptase (MA5-11711) antibodies were purchased from Thermo Scientific Pierce Antibodies. Human IgE, anti-IgE antibody (ab75673) and anti-MIF antibody (ab7207) were bought from Abcam. Phycoerytrin (PE)-labeled anti-CD34 antibody (HPCA-2) and PE-IgG isotype control were purchased from Becton Dickinson (BD). Anti-human FcεRI-FITC and anti-human CD117-PE were purchased from eBioscience. SPI Bio histamine EIA kit was obtained from Bertin Pharma. [3H]-proline was from Perkin-Elmer.

### Cell culture

#### Isolation and characterization of bone marrow CD34+ hematopoietic precursors

The protocol was approved by Ethics Committee of the Skin Disease Research Institute of Chinese Academy of Medical Science and Drum Tower Hospital Ethical Committee. Written informed consent was obtained from the patients. Bone samples were obtained from the excision of the ribs from patients treated in the department of thoracic surgery in Drum Tower hospital. Bone marrow was flushed into a 50-ml tube with RPMI-1640 medium. The cells were centrifuged for 10 minutes at 500g. Re-suspend the cells with 5 ml of RPMI-1640, and add the cell suspension slowly on the top of the Percoll solution (1.0739g/ml). Centrifuge the cells for 30 minutes at 700g without brake at 4°C. Extract the white blur layer which contains the mixed cells. Wash the cells twice with cold PBS. Then re-suspend the cells in the medium (RPMI-1640 completed with 10% FBS, antibiotics, non-essential amino acids, 10μg/ml bovine insulin and 2mM L-glutamine) and culture in a humidified CO_2_ (5%) incubator at 37°C for at least 3 days. The floating cells were then used in the following experiments. CD34+ hematopoietic precursors were isolated from the floating cell mixture using CD34 MicroBead Kit (Miltenyi Biotec, Bergisch Gladbach, Germany) according to the manufactory instruction. Two immunomagnetic cycles were performed to enrich the desired cells.

#### Differentiation of human mast cells from CD34+ hematopoietic precursors

Mast cell medium for human (for differentiation and maintaining of mast cells) was prepared by mixing IMDM with penicillin-streptomycin solution, 2mM L-glutamine, 1mM sodium pyruvate, 1% nonessential amino acid, 10% fetal bovine serum, Human IL-6 (50 ng/ml final concentration), Human SCF (100 ng/ml) and Human IL-3 (5 ng/ml). Isolated CD34+ hematopoietic precursors were cultured in the mast cell medium (change medium every 3 days) for at least 3 weeks before they were used in the following experiments.

#### Culture of human dermal fibroblasts

Human dermal fibroblasts, adult (HDFa) were purchased from Invitrogen and cultured in DMEM plus 2% FBS and antibiotics. Cells were placed in a humidified CO_2_ incubator at 37°C.

### Flow cytometry

The cells were incubated with fluorescence-labeled antibodies (appropriately choose and dilute antibodies according to different experimental purposes) at 4°C for 40 minutes in dark. Cells were washed by centrifugation (1300 rpm, 5 min) and resuspended in cold PBS containing 1% of BSA. To detect CD34 molecules, PE-labeled anti-CD34 antibody was used. As for FcεRI/CD117 double staining, both anti-human FcεRI-FITC and anti-human CD117-PE antibodies were used. For all these experiments, appropriate isotope control antibodies were used. Stained cells were analyzed by using flow cytometry with at least 20,000 events recorded during each measurement. For quantification analysis, the positive/negative boundaries were determined by using cells stained with matched isotope control antibodies; the original data were analyzed with FlowJo software; and the same experiments were repeated for at least three times.

### Toluidine blue and immunofluorescence staining

The derived mast cells were loaded on a glass slide and air-dried. Toluidine blue staining was performed as described in the previous report [32]. The immunofluorescence staining was carried out following the previous report [33] using anti-chymase (mouse) and anti-tryptase (mouse) antibodies. DAPI (1μg/ml) was used to stain cell nuclei. The slides were observed and the pictures were captured using fluorescent microscope (Leica DMI3000B).

### Degranulation assay

MCs were washed with in PBS, resuspended in fresh mast cell medium, and plated in the 6-well plate (10^6^ cells/well). The stimulation was initiated by the addition of IgE (100 ng/ml) for 2 h at 37°C, followed by adding anti-IgE (1μg/ml) for another 2 hr [34, 35]. Released histamine in the supernatant was collected 30 min after cell stimulation and assessed by SPI Bio histamine EIA kit [36]. Similarly, MIF in the supernatant was also measured by ELISA.

### Fibroblast proliferation

Human fibroblasts were seeded in 60mm dishes (10^5^ cells/dish) and allowed to adhere overnight. After that, different medium (90% of the original medium for fibroblasts plus 10% of conditioned or un-conditioned medium of mast cells) was added into each group. Mast cell conditioned medium was collected from stimulated (by adding IgE and anti-IgE antibody) or un-stimulated mast cells were used. For certain experiments, an anti-MIF antibody was added into the medium. The number of the cells in each group was counted during each time of subculture.

### Collagen production by fibroblast

HDFa cells treated in the above experiments were used in collagen production assay. The only difference is that in this experiment 50 μg/ml ascorbic acid and [^3^H]-proline (10 μCi/well) were added in the medium. The cells were cultured for 2 days, and secreted [^3^H]-proline-containing collagen in the medium was determined using the methods described in previous reports [37, 38]. Briefly, total proteins in 500 μl medium (including collagenous and non-collagenous) were precipitated on glass fiber filters and counted in a scintillant. Noncollagenous proteins (collagenase-resistant) in 500 μl medium were measured after digestion with collagenase for 2 h at 37°C. Collagenous proteins were calculated by subtracted noncollagenous proteins from the total proteins. The final results were normalized against the number of cells in each group.

### q-RT-PCR

Total RNA was extracted from the cells using Trizol method. CDNA was synthesized by using iScript Advanced cDNA Synthesis Kit (Bio-Rad). PCR reactions were carried out by using SYBR Green PCR Master Mix (Life Technologies). The primers for MIF were 5’-cgcagaaccgctcctaca-3’ and 5’-ttaggcgaaggtggagttg-3’. The primers for β-actin were 5’-tgacggggtcacccacactgt-3’ and 5’- ttgcggtggacgatggaggg-3’. The mRNA level of MIF was normalized against that of β-actin in the same cells.

### Preparation and application of lentivirus

MIF shRNA was synthesized and sub-cloned into pLKO.1 vector. The targeting sequence of the shRNA is: 5’-agcgagccgtgcgcgctctgc-3’ [39]. Lentiviral particles were produced from 293T cells using ViraPower Lentiviral expression kit (Invitrogen). Lenti-virus was applied on CD34+ hematopoietic precursors for 6 hours before it was changed with fresh medium. Cells were selected with puromycin (1μg/ml) for 6 days to enrich transducted cells. After that the selected cells were cultured in puromycin-free medium for another 3 days, and then they were differentiated into mast cells.

### Statistics

Experimental results are shown as the mean ± S.D. Statistical analyses were performed by unpaired Students t test or ANOVA assuming unequal variance using SigmaPlot 11.0 (San Jose, CA USA) unless otherwise indicated. Significance was defined as *p<0.05, ** p<0.01, *** p<0.001 and #p>0.05.

## Results

### Characterization of bone marrow CD34+ hematopoietic precursors

**Fig 1:**
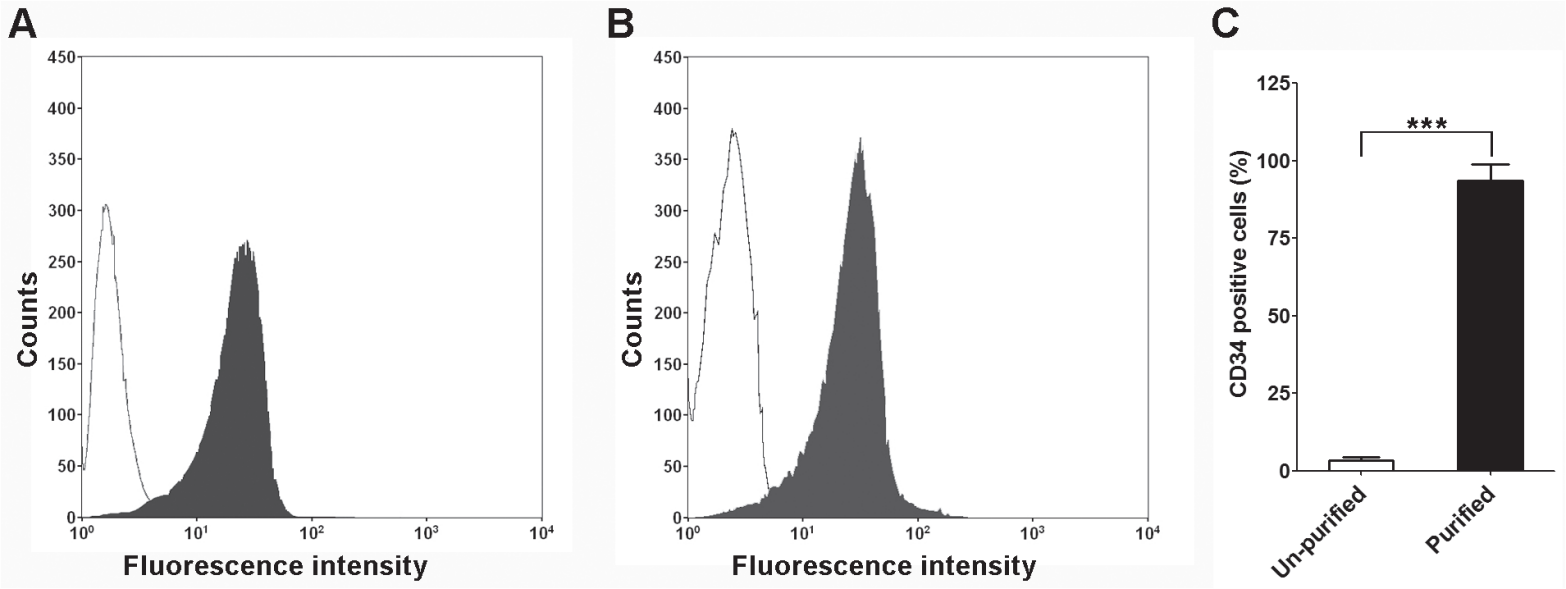
Characterization of bone marrow CD34+ hematopoietic precursors. A. A representative figure of flow cytometry analysis on the purified and un-purified mononuclear cells. PE-labeled anti-CD34 antibody (HPCA-2) was used in this experiment. Histogram shows antibody staining (purified hematopoietic precursors, in dark) and un-staining (un-purified, transparent) cells. B. A representative figure of flow cytometry analysis on purified CD34+ hematopoietic precursors. PE-labeled anti-CD34 antibody (in dark) and its isotype control (transparent) were used in this experiment. C. Quantification analysis of CD34+ population in purified and un-purified cells, mean±SD, n = 3.

In the current study, we were endeavored to isolate CD34+ hematopoietic precursors from human bone marrow and obtain functional mast cells via an in-vitro cell culture differentiation process. As the first step, we successfully isolated CD34+ hematopoietic precursor cells from human bone marrow. As shown in Fig 1A, the purified CD34+ hematopoietic cells were recognized by (PE)-labeled anti-CD34 antibody (dark peak) while most of the non-purified cells were not marked by the same antibody (transparent peak). In order to ensure the specificity of our staining, we also stained the purified cells with (PE)-labeled anti-CD34 antibody or its isotype control. As shown in Fig 1B, the purified CD34+ hematopoietic cells were only marked by anti-CD34 antibody (dark peak) while control isotype failed to bind to these cells. Qualitative analysis showed that about 94% of the purified cells were CD34 positive while only 3.4% of the un-purified cells were CD34 positive (Fig 1C).

### Characterization of mast cells derived from bone marrow CD34+ hematopoietic precursors

**Fig 2:**
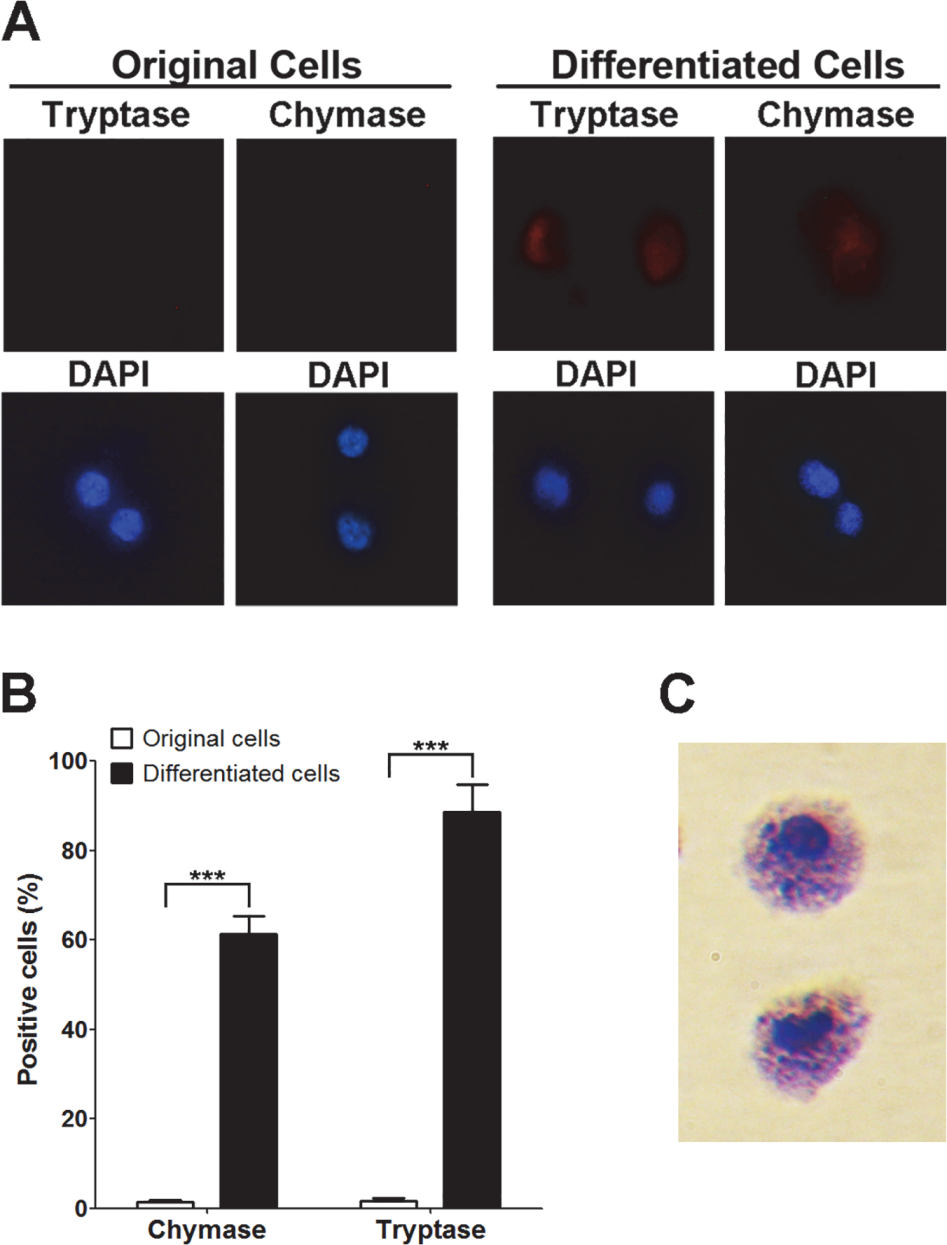
Characterization of mast cells derived from bone marrow CD34+ hematopoietic precursors. A. Immunofluorescence staining of original CD34+ hematopoietic precursors and differentiated mast cells. Anti-tryptase antibody or anti-chymase antibody was used in the experiments. DAPI was used to stain the nucleus of the cells. B. Quantification of experiments described in A, more than 200 cells were counted in each group, mean±SD, n = 3. C. Toluidine blue staining of the derived mast cells.

As the next step, we sought to differentiate CD34+ hematopoietic cells into mast cells in light of previous description of similar methodology [30]. Cytokines, such as human IL-6, SCF and IL-3, were used to stimulate the transition of CD34+ hematopoietic precursors to mast cells. The number of the mast cells gradually increased during differentiation. To test the effectiveness of differentiation cocktail used in this experiment, we chose chymase and tryptase as specific markers for functional mast cells [40]. As shown in Fig 2A and 2B, about 90% of the differentiated cells were positive for tryptase staining while only less than 2% of the non-differentiated cells were positively stained. Similar results were obtained when anti-chymase antibody was used. We observed that about 61% of the differentiated cells were marked by anti-chymase antibody while very few original cells exhibited positive staining. Toluidine staining stands as a classic method to identify mast cells [41]. With this method, we were able to manifest the typical morphological features of mature mast cells in our derived cells, such as enrichment of granules (Fig 2C).

### Degranulation assay of derived MCs

**Fig 3:**
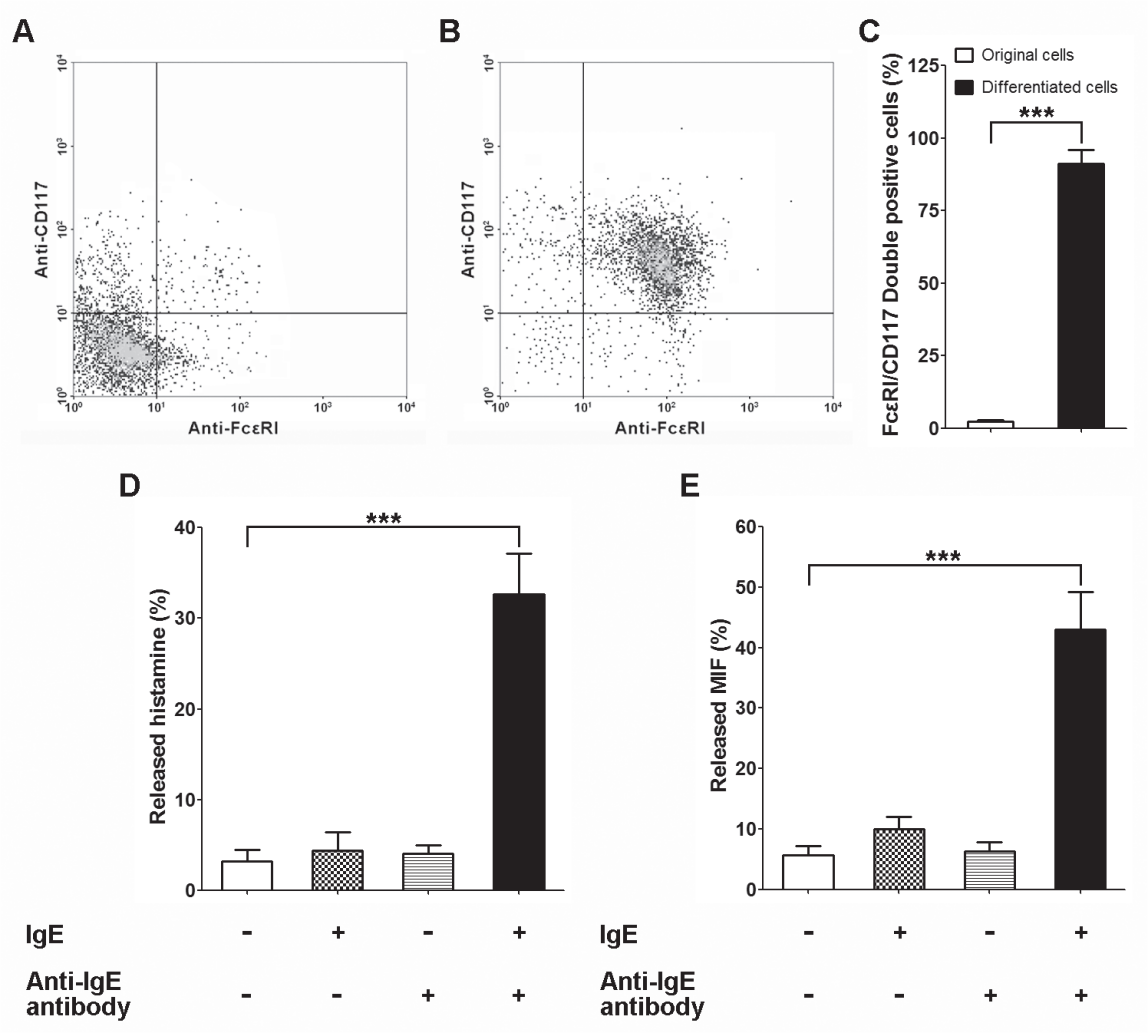
Degranulation assay of derived MCs. A. A representative result of flow cytometry on original CD34+ hematopoietic precursors using anti-FcεRI and anti-CD117 antibodies. B. A representative result of flow cytometry on derived mast cells using anti-FcεRI and anti-CD117 antibodies. C. Quantification analysis of FcεRI/CD117 double positive population in original CD34+ hematopoietic precursors and derived mast cells. D. Mast cell-released histamine in different group was assessed by ELISA assay. E. Mast cell-released MIF was assessed by ELISA assay, mean±SD, n = 3.

As the next step, we felt imperative to test whether the derivative mast cells is functional. Degranulation is a cellular process that releases antimicrobial cytotoxic molecules from secretory vesicles) or granules from inside of the cells. As for mast cells, to induce degranulation, antigens interact with IgE bound to high affinity Fc receptors (FcεRI) on cell surface. Then the mast cells release a series of inflammatory mediators including histamine, proteoglycans, serotonin, and serine proteases from its cytoplasmic granules [42]. In the current study, the expression of FcεRI in differentiated cells was examined by flow cytometry. Since CD117 (stem cell growth factor receptor or called c-kit) plays a vital role in survival, proliferation and differentiation of mast cells, we performed FcεRI/CD117 double staining experiments. As shown in Fig 3A, 3B, and 3C, only a very small portion (<2%) of the un-differentiated cells expressed both molecular markers. On the contrary, over 91% of the differentiated cells were positively stained for both FcεRI and CD117. More importantly, the results meant that the derivative mast cells express FcεRI which is required for degranulation. As a positive control, histamine was found to be secreted by the derivative mast cells (Fig 3D) given the presence of IgE and IgE antibody, which indicates that degranulation function was well preserved in these mast cells. As expected, we also found that MIF was secreted by these mast cells during degranulation (Fig 3E).

### Fibroblast proliferation and collagen synthesis assays in different culture conditions

**Fig 4:**
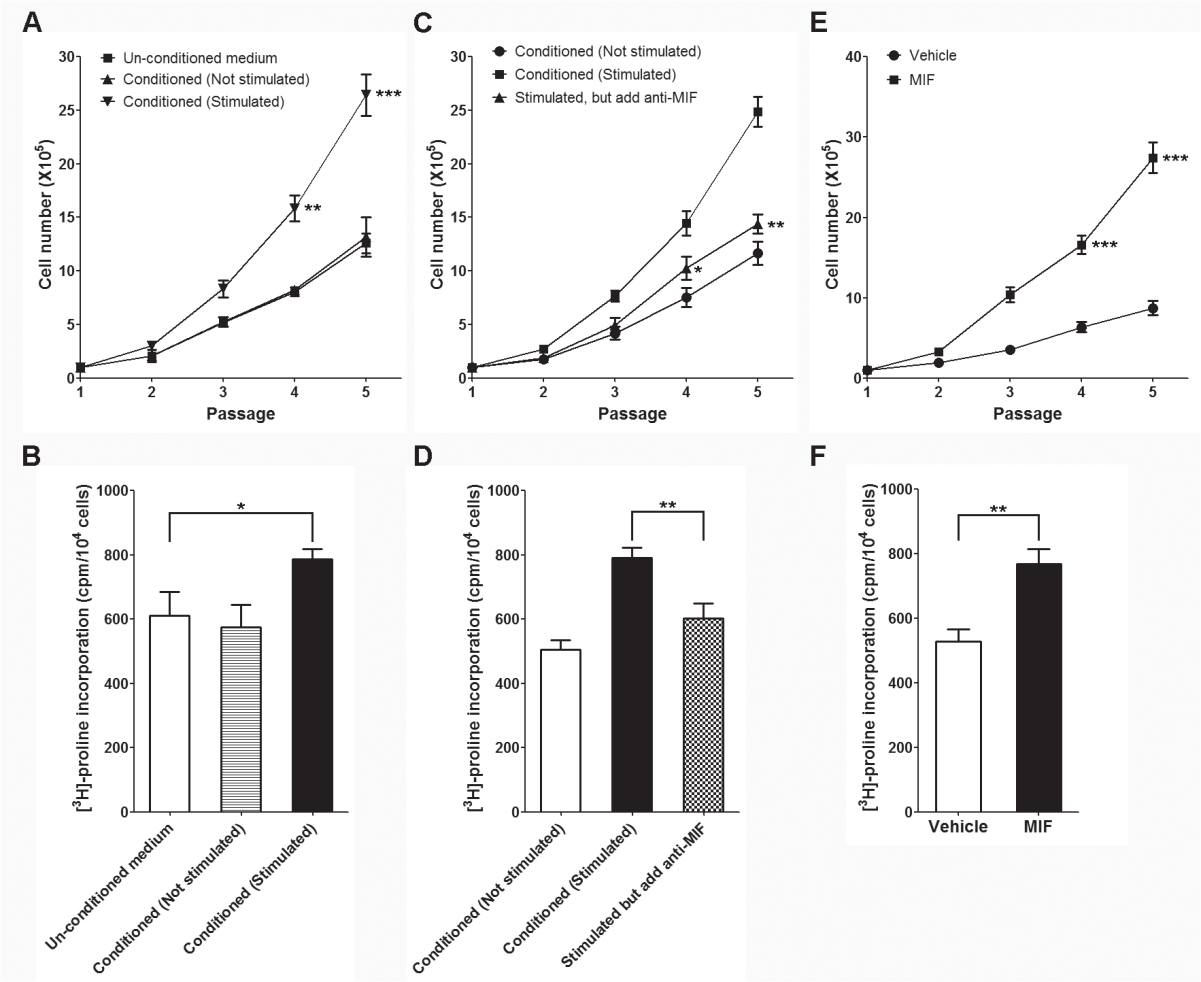
Fibroblast proliferation and collagen synthesis assays in different culture conditions. A and B. The growth curves and collagen synthesis of fibroblast cultured in un-conditioned or conditioned (stimulated or un-stimulated) medium; *P<0.05, **P<0.01, ***P<0.001 compared with un-conditioned group. C and D. The growth curves and collagen synthesis of fibroblast cultured in un-stimulated or stimulated (add anti-MIF antibody or not) medium; *P<0.05, **P<0.01, ***P<0.001 compared with stimulated group without anti-MIF antibody. E and F. The growth curves and collagen synthesis of fibroblast cultured in medium with or without MIF supplement, mean±SD, n = 3.

The above results showed that the derivative mast cells from CD34+ hematopoietic cells possess the appropriate mast cell morphology as well as physiological functions. Subsequently we sought to identify whether derivative mast cells exert positively regulatory effects on fibroblast proliferation and collagen synthesis. As shown in Fig 4A and 4B, conditioned medium of stimulated mast cells (by adding IgE and anti-IgE antibody) significantly increased fibroblast proliferation and collagen synthesis while conditioned medium of un-stimulated mast cells had no such activities. These results implied that stimulators for fibroblast proliferation and collagen synthesis are produced by mast cells and need to be released through degranulation. Since we are interested in the role of MIF in fibrotic diseases, such as scleroderma, we simply added MIF antibody in the conditioned medium of stimulated mast cells. Excitingly, we found that addition of MIF antibody significantly inhibited the profibrogenic activities of conditioned medium of stimulated mast cells (Fig 4C and 4D). These results suggested that MIF is one of the profibrogenic substances released by mast cells during degranulation. To further test our hypothesis, recombinant Human MIF was added into culture medium of HDFa in absence of any conditioned medium of mast cells. As expected, MIF supplement significantly enhances fibroblast growth and collagen synthesis (Fig 4E and 4F). These data strongly suggested that MIF plays an important role to fibrogenic activities of mast cells.

### MIF knockdown inhibits the profibrogenic activity of mast cells

**Fig 5:**
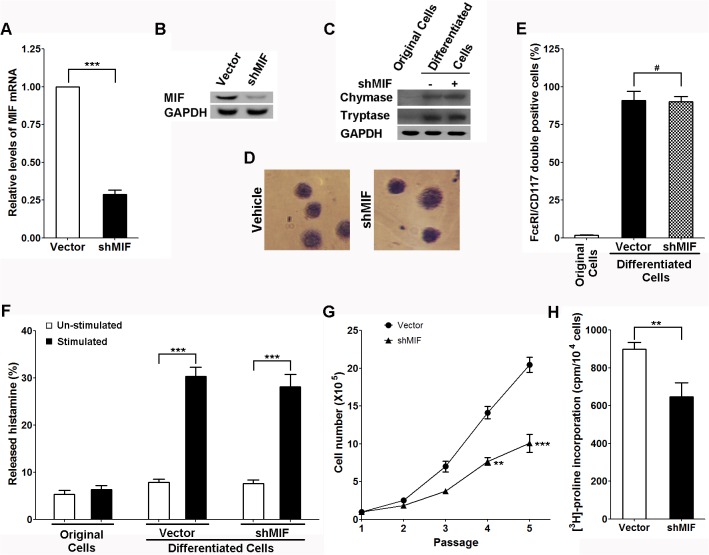
MIF knockdown inhibits the profibrogenic activity of Mast cells. A and B. The mRNA and protein levels of MIF in mast cells derived from CD34+ hematopoietic precursors with or without MIF knockdown. C. Western-blots of chymase, tryptase and GAPDH in original or mast cells (derived from CD34+ hematopoietic precursors with or without MIF knockdown). D. Toluidine blue staining of the mast cells derived from CD34+ hematopoietic precursors with or without MIF knockdown. E. Quantification analysis of FcεRI/CD117 double positive population in original or mast cells (derived from CD34+ hematopoietic precursors with or without MIF knockdown), #P>0.05. F. Histamine release assay of the mast cells derived from CD34+ hematopoietic precursors with or without MIF knockdown. G and H. The growth curves and collagen synthesis of fibroblast cultured in medium conditioned by mast cells with or without MIF knockdown, mean±SD, n = 3.

To further characterize the role of MIF in context of mast cell mediated fibrogenic activities, we first overexpressed shRNA of MIF in the CD34+ hematopoietic precursors and then differentiated these cells to mast cells. As expected, the mast cells derived from MIF-knockdown CD34+ hematopoietic precursors expressed lower levels of MIF mRNA and protein (Fig 5A and 5B). In addition, the results (Fig 5C, 5D, and 5E) revealed that MIF knockdown did not affect the differentiation of mast cells in our experiments manifested by the cellular markers we used. And MIF knockdown did not affect the degranulation function of the mast cells (Fig 5F). However, when the conditioned medium of stimulated MIF-knockdown mast cells was added into HDFa culture medium, it failed to increase fibroblast proliferation (Fig 5G) and collagen synthesis (Fig 5H). These results again strengthened our previous conclusion that MIF greatly contributes to mast cell-mediated fibrogenic activities.

## Discussion

Scleroderma, also known as “systemic sclerosis” is a chronic autoimmune disease which is manifested by hardening (*sclero*) of the skin (*derma*). In the more severe form, it also affects internal organs. As mentioned above, mast cells play a pivotal role in the development of scleroderma. In particular, accumulating evidence indicates that mast cells are required for fibrogenic activities in scleroderma pathophysiology [43].

However, currently there is relatively lack of data on underlying mechanism by which mast cells exert fibrogenic activities. In particular, the lack of mature *in vitro* human mast cell model by which the complicated molecular mechanism can be dissected represents a major obstacle for researchers. In physiological conditions, hematopoietic precursor cells migrated from bone marrow to peripheral tissues where they finally differentiate into mast cells with a panoply of cytokines including stem cell factor and certain interleukins [44]. Previous studies have successfully established mouse mast cell culture derived from mouse bone marrow. Genetically manipulated mouse models provide added-value to identify molecules which are essential for mast cell homeostasis. However, the significant difference between human mast cell and mouse mast cell greatly limits the value of mouse mast cell as a tool in human disease research [45]. These differences include, but are not limited to, Th2 cytokine regulated FcεRI expression [34], responses to prostaglandins [46] and anti-allergic medications [47]. Thus adoption of novel human mast cell culture system seems to be imperative. Classically, human mast cells can be isolated from human skin, lung and peripheral blood [48–50]. However, mast cell from these resources cannot be cultured indefinitely and genetic manipulation on mature mast cells is never easy.

In the current study, we developed a mast cell differentiation protocol from CD34+ hematopoietic cells. We confirmed that the CD34+ hematopoietic cell derived mast cells are normal in both morphology and functions. In addition, we were also able to show the derivative mast cells from CD34+ hematopoietic precursors can be genetically manipulated in this culture system. This protocol provides a solid basis for further identification of novel molecules or cytokines involved in scleroderma pathophysiology.

As one of the mast cell released cytokines, macrophage migration inhibitory factor (MIF) has been considered as an essential regulator of innate immunity traditionally [51]. Emerging evidence has shown that MIF is involved in pathophysiology of scleroderma. In particular, several clinical studies have shown that MIF expression is dysregulated in serum and skin culture derived supernatant [22, 23, 25]. In addition, the role of MIF in scenario of fibrogenic activities were also described in other studies [26–29]. For example, in 2006 Taylor JA *et al*. showed that MIF is implicated in partial bladder outlet obstruction mediated detrusor smooth muscle fibrosis[28]. In line with this notion, a recent clinical study also demonstrated that MIF is up-regulated in both lung tissue and serum of idiopathic pulmonary fibrosis patients [29].

In the current study, we successfully characterized the role of MIF in *in vitro* mast cell culture system. As shown in Fig 4, MIF supplement, as well as mast cell conditioned medium, exerted strong positive regulatory effects on fibrogenic activities. Interestingly, MIF antibody largely dampened the mast cell conditioned medium stimulated fibrogenic activities, which is consistent with the observation in Fig 5 showing the inhibitory effect of MIF knockdown on mast cell mediated fibrogenic activities. Although we should admit there are much more participating cells/molecules other than MIF which exert potent regulatory effects during this process *in vivo* and MIF secreted by cells other than mast cells may also be important, our current *in vitro c*ell culture model is still informative and valuable as it helps to dissect the action of a single cytokine from a fibrogenic network complex.

Taken together, in the current study, we optimized a mast cell culture system by inducing CD34+ hematopoietic cell differentiation. In addition, we showed that MIF plays an essential role in mast cell mediated fibrogenic activities. To the best of our knowledge, this is the first mechanistic report showing the significance of MIF in this context, which constitutes a novel, exciting observation by which potential MIF targeted therapy for scleroderma and other fibrogenic conditions could be developed to a further extent.

## References

[1] Heneberg P. Targeting mast cells and basophils in allergy and beyond: emerging concepts. Current pharmaceutical design. 2011;17(34):3741–3. . 22103844 10.2174/138161211798357818

[2] Mekori YA, Metcalfe DD. Mast cells in innate immunity. Immunological reviews. 2000;173:131–40. . 10719674 10.1034/j.1600-065x.2000.917305.x

[3] Gurish MF, Austen KF. Developmental origin and functional specialization of mast cell subsets. Immunity. 2012;37(1):25–33. 10.1016/j.immuni.2012.07.003 . 22840841

[4] Reber LL, Daubeuf F, Pejler G, Abrink M, Frossard N. Mast cells contribute to bleomycin-induced lung inflammation and injury in mice through a chymase/mast cell protease 4-dependent mechanism. Journal of immunology. 2014;192(4):1847–54. 10.4049/jimmunol.1300875 . 24453258

[5] Jeong DH, Lee GP, Jeong WI, Do SH, Yang HJ, Yuan DW, et al Alterations of mast cells and TGF-beta1 on the silymarin treatment for CCl(4)-induced hepatic fibrosis. World journal of gastroenterology: WJG. 2005;11(8):1141–8. 15754394 10.3748/wjg.v11.i8.1141 PMC4250703

[6] Beghdadi W, Madjene LC, Claver J, Pejler G, Beaudoin L, Lehuen A, et al Mast cell chymase protects against renal fibrosis in murine unilateral ureteral obstruction. Kidney international. 2013;84(2):317–26. 10.1038/ki.2013.98 . 23515052

[7] Nishioka K, Kobayashi Y, Katayama I, Takijiri C. Mast cell numbers in diffuse scleroderma. Archives of dermatology. 1987;123(2):205–8. . 3813593

[8] Irani AM, Gruber BL, Kaufman LD, Kahaleh MB, Schwartz LB. Mast cell changes in scleroderma. Presence of MCT cells in the skin and evidence of mast cell activation. Arthritis and rheumatism. 1992;35(8):933–9. . 1642658 10.1002/art.1780350813

[9] Kalesnikoff J, Galli SJ. New developments in mast cell biology. Nature immunology. 2008;9(11):1215–23. 10.1038/ni.f.216 18936782 PMC2856637

[10] Atamas SP, White B. Cytokine regulation of pulmonary fibrosis in scleroderma. Cytokine & growth factor reviews. 2003;14(6):537–50. . 14563355 10.1016/s1359-6101(03)00060-1

[11] Shiota N, Kakizoe E, Shimoura K, Tanaka T, Okunishi H. Effect of mast cell chymase inhibitor on the development of scleroderma in tight-skin mice. British journal of pharmacology. 2005;145(4):424–31. 10.1038/sj.bjp.0706209 15806109 PMC1576161

[12] Wang HW, Tedla N, Hunt JE, Wakefield D, McNeil HP. Mast cell accumulation and cytokine expression in the tight skin mouse model of scleroderma. Experimental dermatology. 2005;14(4):295–302. 10.1111/j.0906-6705.2005.00315.x . 15810888

[13] Kanbe N, Kurosawa M, Nagata H, Yamashita T, Kurimoto F, Miyachi Y. Production of fibrogenic cytokines by cord blood-derived cultured human mast cells. The Journal of allergy and clinical immunology. 2000;106(1 Pt 2):S85–90. . 10887339 10.1067/mai.2000.106777

[14] Abe M, Yokoyama Y, Amano H, Matsushima Y, Kan C, Ishikawa O. Effect of activated human mast cells and mast cell-derived mediators on proliferation, type I collagen production and glycosaminoglycans synthesis by human dermal fibroblasts. European journal of dermatology: EJD. 2002;12(4):340–6. . 12095878

[15] Garbuzenko E, Nagler A, Pickholtz D, Gillery P, Reich R, Maquart FX, et al Human mast cells stimulate fibroblast proliferation, collagen synthesis and lattice contraction: a direct role for mast cells in skin fibrosis. Clinical and experimental allergy: journal of the British Society for Allergy and Clinical Immunology. 2002;32(2):237–46. . 11929488 10.1046/j.1365-2222.2002.01293.x

[16] Denton CP, Abraham DJ. Transforming growth factor-beta and connective tissue growth factor: key cytokines in scleroderma pathogenesis. Current opinion in rheumatology. 2001;13(6):505–11. . 11698729 10.1097/00002281-200111000-00010

[17] Matsumoto T, Wada A, Tsutamoto T, Ohnishi M, Isono T, Kinoshita M. Chymase inhibition prevents cardiac fibrosis and improves diastolic dysfunction in the progression of heart failure. Circulation. 2003;107(20):2555–8. 10.1161/01.CIR.0000074041.81728.79 . 12742989

[18] Kakizoe E, Shiota N, Tanabe Y, Shimoura K, Kobayashi Y, Okunishi H. Isoform-selective upregulation of mast cell chymase in the development of skin fibrosis in scleroderma model mice. The Journal of investigative dermatology. 2001;116(1):118–23. 10.1046/j.1523-1747.2001.00165.x . 11168806

[19] Ozbilgin MK, Inan S. The roles of transforming growth factor type beta3 (TGF-beta3) and mast cells in the pathogenesis of scleroderma. Clinical rheumatology. 2003;22(3):189–95. 10.1007/s10067-003-0706-5 . 14505209

[20] Santiago B, Gutierrez-Canas I, Dotor J, Palao G, Lasarte JJ, Ruiz J, et al Topical application of a peptide inhibitor of transforming growth factor-beta1 ameliorates bleomycin-induced skin fibrosis. The Journal of investigative dermatology. 2005;125(3):450–5. 10.1111/j.0022-202X.2005.23859.x . 16117784

[21] Chen H, Centola M, Altschul SF, Metzger H. Characterization of gene expression in resting and activated mast cells. The Journal of experimental medicine. 1998;188(9):1657–68. 9802978 10.1084/jem.188.9.1657 PMC2212524

[22] Selvi E, Tripodi SA, Catenaccio M, Lorenzini S, Chindamo D, Manganelli S, et al Expression of macrophage migration inhibitory factor in diffuse systemic sclerosis. Annals of the rheumatic diseases. 2003;62(5):460–4. 12695161 10.1136/ard.62.5.460 PMC1754538

[23] Wu SP, Leng L, Feng Z, Liu N, Zhao H, McDonald C, et al Macrophage migration inhibitory factor promoter polymorphisms and the clinical expression of scleroderma. Arthritis and rheumatism. 2006;54(11):3661–9. 10.1002/art.22179 . 17075815

[24] Bossini-Castillo L, Simeon CP, Beretta L, Vonk MC, Callejas-Rubio JL, Espinosa G, et al Confirmation of association of the macrophage migration inhibitory factor gene with systemic sclerosis in a large European population. Rheumatology. 2011;50(11):1976–81. 10.1093/rheumatology/ker259 . 21875883

[25] Becker H, Willeke P, Schotte H, Domschke W, Gaubitz M. Macrophage migration inhibitory factor may contribute to vasculopathy in systemic sclerosis. Clinical rheumatology. 2008;27(10):1307–11. 10.1007/s10067-008-0960-7 . 18618071

[26] Tanino Y, Makita H, Miyamoto K, Betsuyaku T, Ohtsuka Y, Nishihira J, et al Role of macrophage migration inhibitory factor in bleomycin-induced lung injury and fibrosis in mice. American journal of physiology Lung cellular and molecular physiology. 2002;283(1):L156–62. 10.1152/ajplung.00155.2001 . 12060572

[27] Hori Y, Sato S, Yamate J, Kurasaki M, Nishihira J, Hosokawa T, et al Immunohistochemical study of macrophage migration inhibitory factor in rat liver fibrosis induced by thioacetamide. European journal of histochemistry: EJH. 2003;47(4):317–24. . 14706927

[28] Taylor JA, Zhu Q, Irwin B, Maghaydah Y, Tsimikas J, Pilbeam C, et al Null mutation in macrophage migration inhibitory factor prevents muscle cell loss and fibrosis in partial bladder outlet obstruction. American journal of physiology Renal physiology. 2006;291(6):F1343–53. 10.1152/ajprenal.00144.2006 . 16835407

[29] Bargagli E, Olivieri C, Nikiforakis N, Cintorino M, Magi B, Perari MG, et al Analysis of macrophage migration inhibitory factor (MIF) in patients with idiopathic pulmonary fibrosis. Respiratory physiology & neurobiology. 2009;167(3):261–7. 10.1016/j.resp.2009.05.004 . 19464392

[30] Kovarova M, Latour AM, Chason KD, Tilley SL, Koller BH. Human embryonic stem cells: a source of mast cells for the study of allergic and inflammatory diseases. Blood. 2010;115(18):3695–703. 10.1182/blood-2009-08-237206 20200352 PMC2865868

[31] Kovarova M, Koller B. Differentiation of mast cells from embryonic stem cells. Current protocols in immunology / edited by John E Coligan [et al]. 2012;Chapter 22:Unit 22F 10 1–6. 10.1002/0471142735.im22f10s97 . 22470136

[32] Metcalfe DD. Isolation of tissue mast cells. Current protocols in immunology / edited by John E Coligan [et al]. 2001;Chapter 7:Unit 7 25. 10.1002/0471142735.im0725s01 18432836 PMC2929597

[33] Tu Z, Aird KM, Bitler BG, Nicodemus JP, Beeharry N, Xia B, et al Oncogenic RAS regulates BRIP1 expression to induce dissociation of BRCA1 from chromatin, inhibit DNA repair, and promote senescence. Developmental cell. 2011;21(6):1077–91. 10.1016/j.devcel.2011.10.010 22137763 PMC3241855

[34] Kraft S, Kinet JP. New developments in FcepsilonRI regulation, function and inhibition. Nature reviews Immunology. 2007;7(5):365–78. 10.1038/nri2072 . 17438574

[35] Mirzahosseini A, Dalmadi B, Csutora P. Histamine receptor H4 regulates mast cell degranulation and IgE induced FcepsilonRI upregulation in murine bone marrow-derived mast cells. Cellular immunology. 2013;283(1–2):38–44. 10.1016/j.cellimm.2013.05.006 . 23850674

[36] Takeshita K, Sakai K, Bacon KB, Gantner F. Critical role of histamine H4 receptor in leukotriene B4 production and mast cell-dependent neutrophil recruitment induced by zymosan in vivo. The Journal of pharmacology and experimental therapeutics. 2003;307(3):1072–8. 10.1124/jpet.103.057489 . 14551291

[37] Postlethwaite AE, Smith GN, Mainardi CL, Seyer JM, Kang AH. Lymphocyte modulation of fibroblast function in vitro: stimulation and inhibition of collagen production by different effector molecules. Journal of immunology. 1984;132(5):2470–7. . 6609200

[38] Cairns JA, Walls AF. Mast cell tryptase stimulates the synthesis of type I collagen in human lung fibroblasts. The Journal of clinical investigation. 1997;99(6):1313–21. 10.1172/JCI119290 9077541 PMC507947

[39] Ohta S, Misawa A, Fukaya R, Inoue S, Kanemura Y, Okano H, et al Macrophage migration inhibitory factor (MIF) promotes cell survival and proliferation of neural stem/progenitor cells. Journal of cell science. 2012;125(Pt 13):3210–20. 10.1242/jcs.102210 . 22454509

[40] Walls AF, Amalinei C. Detection of mast cells and basophils by immunohistochemistry. Methods in molecular biology. 2014;1192:117–34. 10.1007/978-1-4939-1173-8_9 . 25149488

[41] Tas J. Microspectrophotometric detection of heparin in young and adult rat mast cells, human mast cells and human basophilic granulocytes stained metachromatically with toluidine blue O. Acta histochemica Supplementband. 1977;Suppl 18:95–100. . 95796

[42] Pawankar R. Mast cells as orchestrators of the allergic reaction: the IgE-IgE receptor mast cell network. Current opinion in allergy and clinical immunology. 2001;1(1):3–6. . 11964662 10.1097/01.all.0000010977.11360.f4

[43] Yukawa S, Yamaoka K, Sawamukai N, Shimajiri S, Saito K, Tanaka Y. Involvement of mast cells in systemic sclerosis. Nihon Rinsho Men'eki Gakkai kaishi = Japanese journal of clinical immunology. 2010;33(2):81–6. . 20453443 10.2177/jsci.33.81

[44] Galli SJ, Tsai M, Piliponsky AM. The development of allergic inflammation. Nature. 2008;454(7203):445–54. 10.1038/nature07204 18650915 PMC3573758

[45] Saito H, Hatake K, Dvorak AM, Leiferman KM, Donnenberg AD, Arai N, et al Selective differentiation and proliferation of hematopoietic cells induced by recombinant human interleukins. Proceedings of the National Academy of Sciences of the United States of America. 1988;85(7):2288–92. 3258425 10.1073/pnas.85.7.2288 PMC279976

[46] Nguyen M, Solle M, Audoly LP, Tilley SL, Stock JL, McNeish JD, et al Receptors and signaling mechanisms required for prostaglandin E2-mediated regulation of mast cell degranulation and IL-6 production. Journal of immunology. 2002;169(8):4586–93. . 12370397 10.4049/jimmunol.169.8.4586

[47] Okayama Y, Benyon RC, Rees PH, Lowman MA, Hillier K, Church MK. Inhibition profiles of sodium cromoglycate and nedocromil sodium on mediator release from mast cells of human skin, lung, tonsil, adenoid and intestine. Clinical and experimental allergy: journal of the British Society for Allergy and Clinical Immunology. 1992;22(3):401–9. . 1375128 10.1111/j.1365-2222.1992.tb03102.x

[48] Ishizaka T, Conrad DH, Schulman ES, Sterk AR, Ishizaka K. Biochemical analysis of initial triggering events of IgE-mediated histamine release from human lung mast cells. Journal of immunology. 1983;130(5):2357–62. . 6187853

[49] Lawrence ID, Warner JA, Cohan VL, Hubbard WC, Kagey-Sobotka A, Lichtenstein LM. Purification and characterization of human skin mast cells. Evidence for human mast cell heterogeneity. Journal of immunology. 1987;139(9):3062–9. . 2444649

[50] Radinger M, Jensen BM, Kuehn HS, Kirshenbaum A, Gilfillan AM. Generation, isolation, and maintenance of human mast cells and mast cell lines derived from peripheral blood or cord blood. Current protocols in immunology / edited by John E Coligan [et al]. 2010;Chapter 7:Unit 7 37. 10.1002/0471142735.im0737s90 20814942 PMC2923748

[51] Calandra T, Roger T. Macrophage migration inhibitory factor: a regulator of innate immunity. Nature reviews Immunology. 2003;3(10):791–800. 10.1038/nri1200 . 14502271 PMC7097468

